# Antioxidant Activity of *Auricularia auricula* Polysaccharides with Different Molecular Weights and Cytotoxicity Difference of Polysaccharides Regulated CaOx to HK-2 Cells

**DOI:** 10.1155/2023/9968886

**Published:** 2023-12-23

**Authors:** Bao-Li Heng, Fan-Yu Wu, Jing-Hong Liu, Jian-Ming Ouyang

**Affiliations:** ^1^Yingde Center, Institute of Kidney Surgery, Jinan University, Guangzhou, Guangdong, China; ^2^Department of Urology, People's Hospital of Yingde City, Yingde, China; ^3^Institute of Biomineralization and Lithiasis Research, Jinan University, Guangzhou 510632, China

## Abstract

**Objective:**

This study aimed to investigate the growth of calcium oxalate (CaOx) crystals regulated by *Auricularia auricular* polysaccharides (AAPs) with different viscosity-average molecular weights (*M*_*v*_), the toxicity of AAP-regulated CaOx crystals toward HK-2 cells, and the prevention and treatment capabilities of AAPs for CaOx stones.

**Methods:**

The scavenging capability and reducing capacity of four kinds of AAPs (*M*_*v*_ of 31.52, 11.82, 5.86, and 3.34 kDa) on hydroxyl, ABTS, and DPPH free radicals and their capability to chelate divalent iron ions were detected. AAP-regulated CaOx crystals were evaluated by using zeta potential, thermogravimetric analysis, X-ray diffraction, and scanning electron microscopy. The cytotoxicity of AAP-regulated crystals was evaluated through examination of cell viability, cell death, malondialdehyde (MDA) content, and cell surface hyaluronic acid (HA) expression.

**Results:**

The in vitro antioxidant activities of the four AAPs were observed in the following order: AAP0 < AAP1 < AAP2 < AAP3. Thus, AAP3, which had the smallest *M*_*v*_, had the strongest antioxidant activity. AAPs can inhibit the growth of CaOx monohydrate (COM), induce the formation of CaOx dihydrate (COD), and reduce the degree of crystal aggregation, with AAP3 exhibiting the strongest capability. Cell experiments showed the lowest cytotoxicity in AAP3-regulated CaOx crystals, along with the lowest MDA content, HA expression, and cell mortality. In addition, COD presented less cytotoxicity than COM. Meanwhile, the cytotoxicity of blunt crystals was less than that of sharp crystals.

**Conclusion:**

AAPs, particularly AAP3, showed an excellent antioxidative capability in vitro, and AAP3-regulated CaOx crystals presented minimal cytotoxicity.

## 1. Introduction

The formation of calcium oxalate (CaOx) kidney stones is a complex biological regulatory process [1], and it includes the nucleation, growth, crystallization, and retention of crystals in the kidney [2, 3]. CaOx kidney stones have two main forms: CaOx monohydrate (COM) and CaOx dihydrate (COD). COM likely adheres to the renal wall [4], whereas COD is prone to excretion from the body. Compared with healthy controls, kidney stone patients show higher levels of COM and lower levels of COD in their urine [5].

Although crystal formation is crucial to the initial development of CaOx stones, CaOx crystal growth is slow. Thus, in the typical urine transportation process, the growth of single crystals is insufficient to retain them in the collecting tubule of the kidney [5]. Therefore, the adhesion of CaOx crystals is a key step in the formation of CaOx stones [6], and cell damage is an important cause of adhesion. Many negatively charged molecules or ions, such as bikunin [7], osteopontin [7], hyaluronic acid (HA) [8], annexin A1, and heat shock protein 90 [9], are expressed on the surface of damaged cells. These negatively charged substances can adhere to Ca^2+^ and positively charged CaOx crystals [10].

Glucosaminoglycans (GAGs) are important stone inhibitors in urine. The urine of a normal control group (21.3 ± 10.4 m·g/L) contained significantly higher concentration of GAGs compared with that of kidney stone patients (12.5 ± 6.3 m·g/L) [11]. High amounts of GAGs are rich in sulfate groups (−OSO_3_^−^) and/or carboxyl groups (−COO^−^), which can complex with Ca^2+^ in urine and reduce the concentration of free Ca^2+^ in urine and inhibit the formation of CaOx stones [12].

Natural polysaccharide material resources are extensive [13] and have low toxicity and few side effects [14]; they are also rich in (−OSO_3_^−^) and/or carboxyl groups (−COO^−^). In particular, plant polysaccharides have the same structure and physicochemical properties as GAGs [15]; therefore, they may be used to prevent the formation of CaOx stones. However, the high molecular weight (*M*_*w*_) and high viscosity of natural plant polysaccharides limit their application in medicine [16].

Compared with those of natural polysaccharides, the functional groups of degraded polysaccharides do not change remarkably [17], but their biological activity improves considerably because of their relatively low *M*_*w*_, low viscosity, and good solubility [18]. Sun et al. [19] studied the antioxidant properties of *Porphyra* polysaccharides with different *M*_*w*_; the results show that *Porphyra* polysaccharides with a high *M*_*w*_ (*M*_*w*_ = 2918 kDa) had no evident antioxidant activity, but the degraded polysaccharide fragments (*M*_*w*_ = 256.2, 60.66, 6.55 kDa) exhibited inhibitory effects on oxidative damage, with the following antioxidant activities: 6.55 kDa > 60.66 kDa > 256 kDa. Zhou et al. [20] observed that *λ*-carrageenan samples with different *M*_*w*_ (*M*_*w*_ = 9.3, 15, 140, 240, 650 kDa) can inhibit the growth of S180 and H22 tumors in mice. The polysaccharides with *M*_*w*_ of 9.3 and 15 kDa showed the strongest anti-S180 and anti-H22 tumor activities, with inhibition rates of 66.15% and 68.97%, respectively, which were significantly higher than those of polysaccharides with *M*_*w*_ > 15 kDa (37.64%–57.58% and 23.22%–61.90%). Green tea polysaccharides with different *M*_*w*_ (10.88, 8.16, 4.82, and 2.30 kDa) can reduce the oxidative damage on HK-2 cells induced by oxalic acid, and the strongest activity was observed at *M*_*w*_ of 4.82 kDa [21]. Four degraded fractions of *Porphyra yezoensis* polysaccharides with *M*_*w*_ of 4.02, 12.6, 49.5, and 576.2 kDa can repair HK-2 cells, inhibit crystal adhesion, and promote endocytosis, and the fraction with the lowest *M*_*w*_ exhibited the best biological activity [22].

The fruiting body of *Auricularia auricula* is widely used as food and medicine in East Asia [23]. The polysaccharides extracted from *Auricularia auricula* (AAPs) are the key bioactive components of the fungus [24]. AAPs are composed of a D-glucose residue backbone and various *β*-1,3-branched residue chains, such as glucose, mannose, and xylose [25]. AAPs regulate in vitro immune activity and exhibit antitumor and antioxidation biological activities [26, 27].

In this study, AAPs with *M*_*w*_ of 3.34, 5.86, 11.82, and 31.52 kDa were obtained by degrading an AAP with a *M*_*w*_ of 31.52 kDa. The antioxidant capacity of AAPs in vitro, regulation of CaOx crystals, and toxicity difference and damage mechanism of AAP-regulated CaOx crystals in HK-2 cells were studied to provide reference for the development of green drugs inhibiting stone formation.

## 2. Methods and Materials

### 2.1. Reagents and Materials

#### 2.1.1. Reagents

*Auricularia auricular* polysaccharide (AAP0; molecular weight of 31.52 kDa) was produced by Shaanxi Ciyuan Biological Co., Ltd.

Standard monosaccharides, including inositol, galactose, mannose, glucose, arabinose, rhamnose, fucose, and xylose, were purchased from Sigma Chemical Co. (St. Louis, MO, USA). D_2_O (99.9%) was acquired from Shanghai Macklin Biochemical Co., Ltd. (Shanghai, China). Biotinylated HA sbinding protein (bHABP) was obtained from MERCK (Germany). 4′,6-Diamindino-2-phenylindole (DAPI), propidium iodide (PI), Cell Counting Kit-8 (CCK-8 kit), antifluorescence quenching tablet, and bovine serum albumin were secured from Beyotime Biotechnology Co., Ltd. (Shanghai, China). Fluorescein isothiocyanate-Avidin (FITC-Avidin) was bought from Wuhan Boster Biological Technology Co., Ltd (Hubei, China). DMEM/F12 medium, fetal bovine serum, penicillin-streptomycin antibiotics, and trypsin were purchased from Gibco (USA).

2,2′-Biazobis (3-ethylbenzothiazolin-6-sulfonic acid) diammonium salt (ABTS), trifluoroacetic acid (TFA), ferrozine, ascorbic acid (Vc), K_2_S_2_O_8_, 1, 1-diphenyl-2-trinitrophenylhydrazine (DPPH), K_3_[Fe(CN)_6_], NaBD_4_, H_2_O_2_, Na_2_Ox, and CaCl_2_ were analytically pure and purchased from Shanghai Aladdin Biochemical Technology Co., Ltd. (Shanghai, China). Secondary distilled water was used in the experiment.

#### 2.1.2. Instruments

The instruments used in this study included the following: UV-Vis-NIR spectrophotometer (Cary 5000, Agilent, USA), Fourier transform infrared (FT-IR) spectrometer (EQUINOX 55, Bruker, Germany), nuclear magnetic resonance (NMR) spectrometer (AVANCE NEO 600M, Bruker, Germany), gas chromatography-mass spectrometer (GC-MS, 7890A-5975C, Agilent, USA), X-L environmental scanning electron microscope (ESEM, Philips, Netherlands), X-ray powder diffractometer (D/MAX2400, Rigaku, Japan), thermogravimetric analyzer (TGA/DSC 3+, Mettler Toledo, Switzerland), nanoparticle-size zeta potential analyzer (Zatasizer 300HS, Malvern, UK), laser scanning confocal microscope (LSM 800, Zeiss, Germany), enzymometer (Safire2, Tecan, Switzerland), and inverted fluorescence microscope (DMRA2, Leica, Germany).

### 2.2. Degradation and Characterization of Polysaccharides

#### 2.2.1. Degradation of Polysaccharides

AAP0 was used for AAP degradation during the experiment. A total of 1.20 g AAP0 was weighed accurately and dissolved in 40 mL distilled water at 70°C. When the temperature rose to 90°C, 5 mL 30% H_2_O_2_ was rapidly added to degrade AAP0 for 2 h. Then, the pH of the polysaccharide solution was adjusted to 7.0 using 2 mol/L NaOH solution, and the polysaccharide solution was concentrated to about 1/3 of the original volume at 60°C under reduced pressure. Next, three times the volume of anhydrous ethanol was added to precipitate polysaccharides. The precipitate was stored in the refrigerator at 4°C overnight, pumped and filtered, washed twice with anhydrous ethanol, and dried in vacuum to obtain the degraded polysaccharides.  Table 1 shows the degradation conditions of polysaccharides.

#### 2.2.2. Determination of the Molecular Weight of Polysaccharides

The falling time of polysaccharides in the viscometer at 25 ± 0.2°C was measured to calculate the relative viscosity (*η*_*r*_), specific viscosity (*η*_sp_), intrinsic viscosity (*η*), and viscosity-average molecular weight (*M*_*v*_) of polysaccharides. First, the falling times of polysaccharide solution (*T*_*i*_) and deionized water (*T*_0_) were measured using a viscometer. Then, *η*_*r*_ of polysaccharides was calculated using the equation *η*_*r*_=*T*_*i*_/*T*_0_; *η*_sp_ of polysaccharide was calculated using *η*_sp_=*η*_*r*_ − 1. [*η*] was obtained through a one-point method formula [*η*]=[2(*η*_sp_ −  ln *η*_*r*_)]^1/2^/*c*, where *c* refers to the concentration of sample to be measured. *M*_*v*_ of polysaccharide was calculated using the Mark–Houwink empirical equation: [*η*] = *κM*_*v*_^*α*^, where *κ* and *α* are parameters of the empirical equation and depend on polymer morphology, solvent, and temperature.

#### 2.2.3. Determination of −COOH Content of Polysaccharides

Referring to literature [28], the −COOH content of four polysaccharides was detected via conductivity titration, and the concentration of standard NaOH solution was *c*(NaOH) = 0.04616 mol/L.

#### 2.2.4. FT-IR Spectrum Detection in Polysaccharides

A total of 2.0 mg dried polysaccharide sample was ground using 200 mg KBr, tableted, and scanned in the range of 4000^−400^ cm^−1^.

#### 2.2.5. ^1^H NMR and ^13^C NMR Spectra of Polysaccharides

A total of 40 mg completely dried polysaccharides were weighed, added to a nuclear magnetic tube containing 0.5 mL D_2_O, and then completely dissolved for detection.

#### 2.2.6. Determination of Monosaccharide Components via GC-MS

Exactly 10 mg polysaccharides were dissolved in 2 mL TFA (2.5 mol/L) and hydrolyzed at 121°C for 90 min. The solution was evaporated and concentrated to dryness using N_2_ flow. The mixed solution of alcohol and water (1 : 1) was added, and the mixture was evaporated again to dryness. The filter residue was dissolved in 1 mL NH_4_OH (2 mol/L) and 1 mL freshly prepared NaBD_4_ (1 mol/L), reacted at room temperature for 2.5 h, evaporated to dryness under a nitrogen stream, evaporated and concentrated twice using 5% acetic acid in methanol, and then evaporated and concentrated twice using methanol to remove boric acid. A total of 1 mL acetic anhydride was acetylated at 100°C for 2.5 h, and the product was extracted using dichloromethane, washed with distilled water, and dried for GC-MS analysis.

### 2.3. Antioxidant Activity of AAPs

#### 2.3.1. Hydroxyl Radical (·OH) Scavenging

The scavenging capacity of ·OH was determined through the phenanthroline method, and ·OH free radicals were generated through Fenton reaction of the H_2_O_2_/Fe^2+^ system. The total reaction can be expressed as Fe^2+^ + H_2_O_2_ ⟶ Fe^3+^ + OH^−^ + ·OH. The maximum absorption peak at 536 nm disappeared after the phenanthroline-Fe^2+^ aqueous solution was oxidized by ·OH radical to phenanthroline-Fe^3+^. According to the above principle, the oxidation of ·OH radicals was reflected by the change in A536, and the decrease in A536 reflected the increased capability of the sample to scavenge ·OH radicals.

The specific method is as follows: Vc was used as a positive control group, and 2 mL FeSO_4_ solution (2.5 mmol/L), 2 mL phenanthroline solution (2.5 mmol/L), 2 mL PBS with pH = 7.4, and 2 mL H_2_O_2_ (20 mmol/L) were added successively, followed by the addition of 2 mL polysaccharide solution (0.15–3.0 mg/mL). The absorbance at 536 nm (*A*_*i*_) was detected after incubation at 37°C for 90 min. In the blank group, distilled water was used to replace the sample, and the other steps were the same as those used for the product group. The measured absorbance value was *A*_0_. The control group had distilled water instead of H_2_O_2_, the remaining steps as the blank group were used, and the measured absorbance was *A*_*j*_. The average for each group was obtained using three times of parallel operations. The scavenging rate of ·OH radical was calculated using the following formula:

(1) $$\begin{array}{c} \cdot \text{OH scavenging rate }\left(\%\right)=\left[\frac{\left(A_{i}-A_{0}\right)}{\left(A_{j}-A_{0}\right)}\right]\times 100. \end{array}$$

In the formula, *A*_*i*_, *A*_*j*_, and *A*_0_ refer to the absorbances of the sample solution, control group (distilled water instead of H_2_O_2_), and blank group (distilled water instead of sample), respectively.

#### 2.3.2. Scavenging of DPPH Free Radicals

The DPPH solution with a concentration of 0.4 mmol/L was prepared using anhydrous ethanol as the solvent, and the reserve solutions of polysaccharides and Vc with a concentration of 3.00 mg/mL were prepared using distilled water as the solvent. The polysaccharide reserve solution was diluted to 0.15, 0.50, 0.80, 1.00, 2.00, and 3.00 mg/mL before use, and Vc was used as a positive control. A total of 2 mL polysaccharide solution or Vc was added, followed by the addition of 6 mL DPPH solution. The resulting solution was mixed, and the background was replaced by anhydrous ethanol of the same volume using the above method. Each sample solution was paralleled to three duplicate holes. The sample absorbance was measured at 517 nm after incubation at 37°C for 30 min in the dark. The scavenging rate of DPPH free radical was calculated using the following formula:

(2) $$\begin{array}{c} \text{DPPH scavenging rate }\left(\%\right)=\left[1\;-\;\frac{\left(A_{i}-A_{j}\right)\;}{A_{0}}\right]\times 100, \end{array}$$

where *A*_*i*_, *A*_*j*_, and *A*_0_ denote the absorbance of the sample solution, background absorbance of the sample solution, and absorbance of the blank control.

#### 2.3.3. Scavenging of ABTS Free Radicals

The ABTS radical-scavenging capability of AAPs was measured in accordance with the literature study [23]. ABTS methanol solution was mixed with potassium persulfate (2.45 mmol/L) to prepare the ABTS reserve solution (7 mmol/L). The stock solution was stored overnight at room temperature in the dark. Then, the solution was diluted with methanol and mixed with 0.1 mL AAP solution (0.15–3.00 mg/mL). Next, the mixture was allowed to react at room temperature for 6 min. Absorbance was measured at 734 nm, with Vc as the positive control. The scavenging capability of ABTS free radicals was determined using the following formula:

(3) $$\begin{array}{c} \text{ABTS scavenging rate }\left(\%\right)=\left[1-\;\frac{\left(A_{i}-A_{j}\right)}{A_{0}}\right]\times 100, \end{array}$$

where *A*_*i*_, *A*_*j*_, and *A*_0_ refer to the absorbance of the sample (sample and ABTS), control (without sample), and background (without ABTS) solutions, respectively.

#### 2.3.4. Reducing Capacity

The reducing capacity of AAPs was slightly modified following the method described by Wu et al. [23]. A total of 2 mL AAP solution (0.15–3.00 mg/mL) was added to 2 mL PBS (pH = 6.6) and 2 mL potassium ferricyanide solution (1%), and the mixture was incubated at 50°C for 20 min. Then, 2 mL 10% trichloroacetic acid solution was added to terminate the reaction, and the obtained mixture was centrifuged at 4000 rpm for 10 min. Exactly 2 mL supernatant was mixed with 2 mL distilled water and 0.5 mL 0.1% FeCl_3_, and the mixture was allowed to stand for 10 min. The absorbance of the mixture was measured at 700 nm, and Vc was used as a positive control.

#### 2.3.5. Fe^2+^-Chelating Capability

Ferrozine can form a red complex with Fe^2+^, and its maximum absorption wavelength is 562 nm. However, in the presence of chelating agents, the formation of complexes is disrupted, and the red color becomes lighter [29].

The mixture of 2 mL AAPs of different concentrations (0.15–3.00 mg/mL), FeCl_2_ (0.1 mL, 2 mmol/L), and phenazine (0.4 mL, 5 mmol/L) was fully shaken and incubated at room temperature for 10 min. The mixed solution without polysaccharides was used as the blank group, and its absorbance was measured at 562 nm. The capability of different AAPs to chelate Fe^2+^ was calculated using the following formula:

(4) $$\begin{array}{c} {\text{Fe}}^{2+}-\text{chelating capability }\left(\%\right)=\left[\frac{\left(A_{\text{blank group}}-A_{\text{sample}}\right)}{A_{\text{blank group}}}\right]\times 100. \end{array}$$

### 2.4. Growth and Characterization of CaOx Crystals Regulated by Polysaccharides

#### 2.4.1. Crystal Growth

A total of 40 mL 22 mmol/L CaCl2 solution and AAPs with a final concentration of 1.0 g/L was added to a beaker, followed by the addition of distilled water to 48 mL, and stirred for 5 min. Then, 40 mL 22 mmol/L Na2Ox was added to attain an 88 mL final volume of the system, at which point *c*(AAPs) = 0.6 g/L and *c*(Ca^2+^) = *c*(Ox2^−^) = 10 mmol/L. After the reaction at 37°C for 10 min, CaOx crystals were obtained through centrifugation, cleaning, and drying for 2 h.

#### 2.4.2. XRD Characterization

The K-value method was used to calculate the relative percentages of COM and COD in CaOx [30]. The relative mass percentage of COD was computed as follows:

(5) $$\begin{array}{c} \text{COD}\%=\frac{I_{\text{COD}}}{I_{\text{COD}}+I_{\text{COM}}}\times 100, \end{array}$$

where *I*_COM_ and *I*_COD_ refer to the intensity of the main diffraction peak planes of COM ($\bar{1}01$) and COD (200), respectively.

#### 2.4.3. SEM Detection

A small amount of synthetic CaOx crystals were dispersed in anhydrous ethanol, and the samples were placed on the slide after ultrasonication. After drying, gold was sprayed, and the crystals were observed via SEM.

#### 2.4.4. Zeta Potential Detection

CaOx crystal suspension with a concentration of 200 *µ*g/mL was prepared, and the zeta potential was measured using a zeta potential analyzer after ultrasonication for 10 min.

#### 2.4.5. Thermogravimetric Analysis

A total of 5 mg samples were placed in an Al_2_O_3_ crucible and detected in a TGA/DSC 3+ thermogravimetric analyzer.

### 2.5. Cytotoxicity of CaOx Crystals Regulated by Polysaccharides

#### 2.5.1. Cell Culture

Human renal proximal tubular epithelial (HK-2) cells (Shanghai Cell Bank, Chinese Academy of Sciences, China) were cultured in DMEM/F12 medium (containing 10% FBS and 1% penicillin-streptomycin antibiotics) at 37°C and 5% CO_2_.

#### 2.5.2. Detection of Cytotoxicity Using CCK-8

Cells were seeded in 96-well plates at a density of 1.0 × 10^5^ cells/mL (100 *μ*L/well) and incubated at 37°C with 5% CO_2_ for 24 h. The cells were washed with PBS and divided into groups for different treatments. The cells used in the experiment were divided into the following three groups: (1) normal control group: added with serum-free medium; (2) DC control group: added with 200 *μ*g/mL CaOx crystals and regulated in the absence of polysaccharide; and (3) crystal AAP-regulated damage group: CaOx crystal-regulated AAPs added at 200 *μ*g/mL. After incubation for 6 h, 10 *μ*L CCK-8 reagent was added to each well to detect its OD value at 450 nm. The formula is as follows:

(6) $$\begin{array}{c} \text{Cell viability }\left(\%\right)=\left[\frac{\left(\text{OD value of treatment group}\right)}{\left(\text{OD value of control group}\right)}\right]\times 100. \end{array}$$

#### 2.5.3. Detection of Cell Status Using PI

Cells were seeded in six-well plates at 1 × 10^5^ cells/mL (1 mL/well). The pretreatment and grouping of cells were the same as those in Section 2.5.2. After incubation for 6 h, the old culture medium was removed, and the cells were washed twice with PBS and added dropwise with 100 *μ*L PI solution (4 *μ*mol/L). After incubation at 37°C for 5 min, the cells were washed with PBS preheated to room temperature for 3 times. A total of 300 *μ*L PBS was added to each well to observe the PI fluorescence intensity under a fluorescence microscope.

#### 2.5.4. Detection of MDA Content

Pretreatment and grouping of cells were the same as those in Section 2.5.3. After incubation for 6 h, 0.1 mL lysate was added as a blank control, 0.1 mL standard product (series concentration) was added to determine the standard curve, 0.1 mL sample was added for determination, and then 0.2 mL MDA working solution was added for detection. After mixing, the boiling water bath was heated for 15 min, the water bath was cooled to room temperature, and the mixture was centrifuged at 1000 g for 10 min. A total of 200 *μ*L supernatant was added to the 96-well plate, and the absorbance was measured at 532 nm using a laser scanning confocal microscope.

#### 2.5.5. Qualitative and Quantitative Detection of HA Expression

Cells were seeded in a confocal dish at 1.0 × 10^5^ cells/mL, with 1 mL/well. Pretreatment and grouping of cells were the same as those in Section 2.5.3. After incubation for 6 h, the cells were washed twice with PBS, fixed with a fixative for 20 min, and washed thrice with PBS. Then, 100 *μ*L 5 *μ*g/mL bHABP solution was added, and the mixture was incubated overnight at 4°C. Afterward, the cells were washed thrice with PBS, incubated with 100 *μ*L FITC-Avidin for 1 h, and washed thrice again with PBS. DAPI staining solution was added for 4 min, and the cells were washed thrice with PBS. Finally, the cells were sealed with antiquenching agent and observed under a confocal microscope. The expression of HA was confirmed by green cell surface, and the nucleus was stained blue by DAPI.

Quantitative analysis of HA: The fluorescence intensity of HA was determined using the Axiovision software (ZEISS, Germany) connected to an instrument. HA in 100 cells was quantitatively detected for each sample, and the average value was obtained.

### 2.6. Statistical Analysis

All data are expressed as the mean ± standard deviation ($\bar{x}$ ± SD) of three parallel groups. IBM SPSS Statistics 26 software was used for one-way analysis of variance. *p* > 0.05 indicates no significant difference; 0.01 < *p* < 0.05 indicates a significant difference; *p* < 0.01 indicates a highly significant difference.

## 3. Results

### 3.1. Degradation and Characterization of AAP0

#### 3.1.1. Degradation of Polysaccharides and Determination of Average Molecular Weight (*M*_*v*_)

AAP0 (*M*_*v*_: 31.52 kDa) was degraded by hydrogen peroxide (H_2_O_2_) at reaction concentrations of 0.3%, 2%, and 8%, and AAP1, AAP2, and AAP3 with *M*_*v*_ of 11.82, 5.86, and 3.34 kDa were obtained (Table 1), respectively. The factors that affected AAP0 degradation included H_2_O_2_ concentration, degradation temperature, and degradation time. In this paper, the degraded polysaccharides with suitable *M*_*v*_ can be obtained by changing the concentration of H_2_O_2_.

#### 3.1.2. Content of −COOH in AAPs

**Figure 1:**
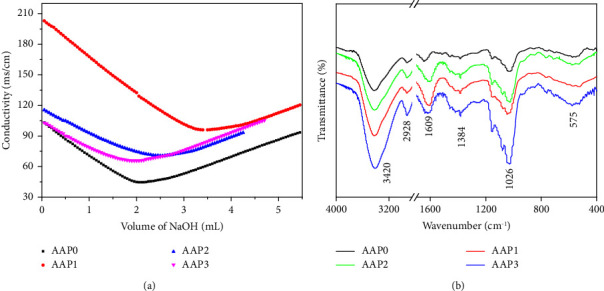
Conductometric titration curve for determination of the content of −COOH in AAPs (a) and FT-IR spectra (b) of AAPs.

The content of −COOH in polysaccharides can be obtained by measuring the conductivity curve of polysaccharides [28]. As shown in Figure 1(a), the −COOH contents of AAP0, AAP1, AAP2, and AAP3 reached 4.28%, 4.54%, 4.63%, and 4.67%, respectively (Table 1). Thus, as *M*_*v*_ of the polysaccharides decreased, the content of −COOH increased slightly. For polysaccharides with a large *M*_*w*_, a part of the −COOH was hidden in the interior of the molecule and was not fully exposed to the aqueous solution. Therefore, AAP0 degradation by H_2_O_2_ had minimal effect on the content of −COOH in polysaccharides.

#### 3.1.3. Fourier Transform Infrared Spectroscopy (FT-IR) Spectra of Polysaccharides before and after Degradation

Figure 1(b) shows the FT-IR spectra of AAPs with different *M*_*v*_. The polysaccharides had almost the same characteristic absorption peaks before and after degradation, which indicates that H_2_O_2_ degradation did not destroy the overall structure of AAP0 [23]. The absorption peak at 1609 cm^−1^ corresponded to the asymmetric-vibration absorption peak of carboxylic acid or uronic acid [23]; the absorption peaks at 1384, 1026, and 575 cm^−1^ corresponded to the shear vibration absorption of C−H, O−H stretching vibration of glycosides, and skeleton vibration peak of polysaccharides, respectively.

#### 3.1.4. ^1^H Nuclear Magnetic Resonance (NMR) and ^13^C NMR Spectra of Polysaccharides

**Figure 2:**
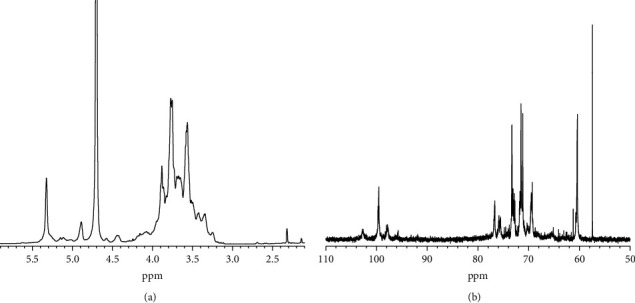
NMR spectra of AAP3. (a) ^1^H NMR spectrum; (b) ^13^C NMR spectrum.

Figure 2(a) shows the ^1^H NMR spectrum of the representative polysaccharide (AAP3). The type of monosaccharide can be determined using the hydrogen proton H-1 of the polysaccharide with a chemical shift at a lower field (*δ* 4.5–5.5 ppm), where the hydrogen proton signal of *δ* 5.32 ppm was ascribed to ⟶4-*α*-Glc*p*-(1⟶) [31]; the signal peak at *δ* 5.12 ppm belonged to (⟶3)-*α*-L-Ara*p*-(1⟶) [32]. The signal peaks at *δ* 4.89 and *δ* 5.14 ppm were assigned to (1⟶)-*α*-D-Man*p* and (1 ⟶ 2)-*α*-D-Man*p*, respectively [33]; the chemical shift at *δ* 4.42 ppm was attributed to (1⟶)-*β*-L-Ara*f* [34].

Figure 2(b) shows the ^13^C NMR spectrum of AAP3, in which the heterocarbon of the polysaccharide was located in the lower field (*δ* 95−110 ppm), and *δ* 102.72 ppm was designated as *α*-D-Man*p*-(1⟶) C-1 [35]; *δ* 99.55 ppm was assigned to *α*-D-Glc*p*-(1⟶) [36] and *δ* 99.20 ppm to (⟶2,4)-*α*-L-Rha*p*-(1⟶) [37]; *δ* 97.92 and *δ* 97.79 ppm were attributed to *α*-D-Glc*p*-(1⟶ and ⟶6)-*α*-D-Glc*p*-(1⟶), respectively [38]; the weak resonance at *δ* 95.69 ppm may be due to the C-1 of *α*-D-Gal*p* [32]; the high-field resonance originated from (1 ⟶ 3)-*α*-L-Ara*p* [32].

**Figure 3:**
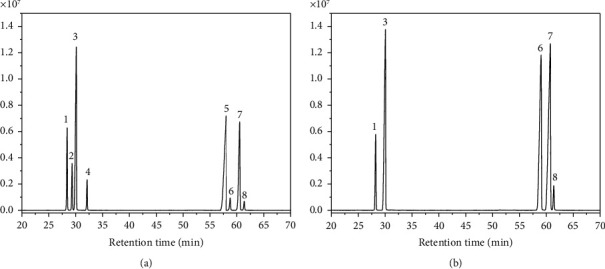
GC-MS spectra of standard monosaccharides (a) and AAP3 (b). (1) Rhamnose; (2) fucose; (3) arabinose; (4) xylose; (5) myo-inositol; (6) mannose; (7) glucose; (8) galactose.

The results of ^1^H NMR and ^13^C NMR spectra show that AAP3 was primarily composed of *α*-D-Man*p*-(1⟶), *α*-D-Glc*p*-(1⟶, ⟶2,4)-*α*-L-Rha*p*-(1⟶), *α*-D-Gal*p*, and (1 ⟶ 3)-*α*-L-Ara*p*, consistent with the results of gas chromatography-mass spectrometry (GC-MS) analysis (Figure 3).

#### 3.1.5. Monosaccharide Composition of AAPs

Figure 3 shows the GC-MS spectrum of AAP3. The retention time of each monosaccharide chromatographic peak in AAP3 (Figure 3(b)) was consistent with the retention times of standard monosaccharide rhamnose, galactose, mannose, glucose, and arabinose (Figure 3(a)), which indicates that AAP3 consisted of the abovementioned monosaccharide components. Analysis of the intensity of each chromatographic peak revealed that the proportions of glucose, mannose, arabinose, galactose, and rhamnose in AAP3 accounted for 61.45%, 17.44%, 14.65%, 3.26%, and 3.20%, respectively (Table 2). These values are close to the proportions reported by Pak et al. [39] (80.02%, 4.78%, 5.64%, 4.78%, and 4.78%).

### 3.2. Antioxidant Activity of AAPs with Different *M*_*w*_

#### 3.2.1. Scavenging Hydroxyl Radicals (·OH)

**Figure 4:**
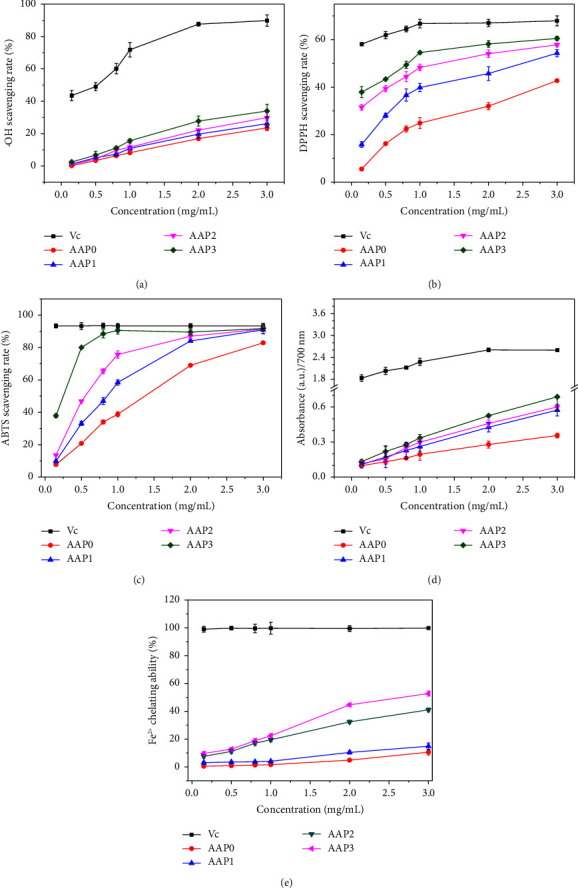
Comparison of antioxidant capacity of different concentrations of AAP0, AAP1, AAP2, and AAP3. (a) ·OH free radical-scavenging ability; (b) DPPH free radical-scavenging ability; (c) ABTS free radical-scavenging ability; (d) reducing capacity; (e) Fe^2+^-chelating capacity.

As shown in Figure 4(a), the capability of AAPs to scavenge ·OH increased with the decrease in *M*_*v*_. At 3.00 mg/mL, the scavenging capability of AAP0 reached 23.55%, whereas that of AAP3 with the smallest *M*_*v*_ was 34.00%.

The scavenging capacity of AAPs for ·OH was highly dose dependent (0.15–3 mg/mL). Notably, the capability of AAP3 to quench ·OH was 2.46% at 0.15 mg/mL, and it increased to 34.00% at 3.00 mg/mL.

Polysaccharides have ·OH scavenging activity, which may be due to their capability to provide hydrogen and bind to free radicals to terminate the free radical chain reaction. In addition, polysaccharides can bind to free radical ions required to free the radical chain reaction, thereby terminating the reaction [40]. However, the exact mechanism by which polysaccharides exert their free radical-scavenging activity remains unclear.

#### 3.2.2. Scavenging of 2,2-Diphenylpicrylhydrazyl (DPPH) Free Radicals

DPPH is a stable free radical in alcohols with a purple color and maximum absorption at 517 nm. When free radical scavengers are present, a single electron of DPPH is captured. As a result, its purple color becomes lighter, and the absorbance decreases. Thus, the DPPH free radical-scavenging capability of the sample can be assessed by detecting the changes in its absorbance.

As shown in Figure 4(b), all degraded polysaccharides showed a stronger DPPH free radical-scavenging activity than AAP0. At 3.00 mg/mL, the scavenging rates of AAP0, AAP1, AAP2, and AAP3 on DPPH free radicals were 42.75%, 54.33%, 57.87%, and 60.53%, respectively.

Similarly, all AAPs showed scavenging capability in a dose-dependent manner at 0.15–3.00 mg/mL. When the concentration of AAP3 increased from 0.15 mg/mL to 3.00 mg/mL, its DPPH scavenging capability increased from 37.94% to 60.53%.

#### 3.2.3. Scavenging of ABTS Free Radicals

ABTS is reduced to form a relatively stable blue-green water-soluble free radical with a maximum absorption at 734 nm. The reaction of antioxidants with ABTS free radicals causes the discoloration of their solution. Therefore, the total antioxidant capacity of the tested substance can be reflected based on the fading condition of the solution.

Figure 4(c) shows the scavenging activity of AAPs on ABTS free radicals. Similar to the results of ·OH and DPPH free radical scavenging, AAP3 had the best scavenging effect. At 3.00 mg/mL, the ABTS-scavenging rates of AAP0, AAP1, AAP2, and AAP3 reached 82.91%, 90.93%, 91.37%, and 91.72%, respectively.

#### 3.2.4. Reducing Capacity

Reducing capacity is used to measure the capability to provide single electrons for antioxidants. The reduction force was measured using the production of Prussian blue Fe_4_ [(Fe(CN)_6_)]_3_. Potassium ferricyanide is reduced by antioxidants to form Prussian blue, which had a maximum absorption peak at 700 nm. Therefore, the reducing power of the sample was positively correlated with absorption.

Figure 4(d) shows the strong to weak reducing capacities of each AAP at the same concentration: AAP3 > AAP2 > AAP1 > AAP0. With the increase in the concentration of the same polysaccharide, the reducing capability was also improved. The reducing capacities of AAP3 were 0.132 and 0.688 at 0.15 and 3 mg/mL, respectively. The reducing capacity is related to the presence of reducing ketones, which exert antioxidant effects by providing a hydrogen atom to destroy the free radical chain [30].

#### 3.2.5. Fe^2+^-Chelating Ability

All four AAPs exhibited Fe^2+^-chelating capability (Figure 4(e)). Compared with the positive control Vc, AAP0 and AAP1 showed weak chelating capabilities toward Fe^2+^, whereas the chelating capabilities of AAP2 and AAP3 toward Fe^2+^ improved considerably because the active groups (−COOH, and so on) of low-*M*_*v*_ polysaccharides were more exposed and had greater degrees of freedom. Therefore, the steric hindrance was small during the chelation with Fe^2+^, and the chelating capability was enhanced [41].

### 3.3. AAP-Regulated CaOx Crystal Growth

#### 3.3.1. Scanning Electron Microscopy (SEM) Observation

**Figure 5:**
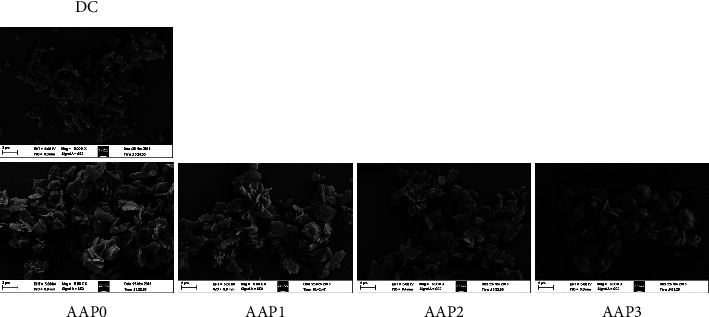
SEM images of the CaOx crystals induced by AAPs with different molecular weights. Polysaccharide concentration: 0.6 g/L.

Figure 5 shows the SEM images of the CaOx crystal induced by AAPs (0.6 g/L). In the absence of polysaccharides, the formed crystals and irregular COM crystals are aggregated. After adding four kinds of AAPs, straw-hat-shaped COD crystals formed. With the decrease in *M*_*v*_ of AAPs, the percentage of COD crystal increased, the aggregation degree of crystals decreased, and the crystal edges became round and blunt.

#### 3.3.2. X-Ray Diffraction (XRD) Characterization

**Figure 6:**
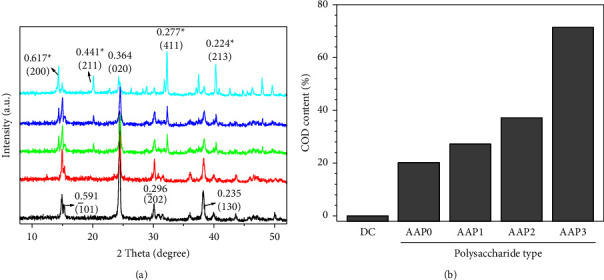
XRD patterns of CaOx crystals induced by four AAPs (a) and COD percentage in crystals (b). Polysaccharide concentration: 0.6 g/L. The crystal faces with asterisk show COD and those without asterisk show COM.

Figure 6(a) shows the XRD patterns of AAP-induced CaOx crystals (0.6 g/L). The diffraction peaks of ($\bar{1}01$), (020), ($\bar{2}02$), and (130) crystal planes of COM appeared at *d* = 0.591, 0.364, 0.296, and 0.235 nm, respectively. The diffraction peaks of the (200), (211), (411), and (213) crystal planes of COD emerged at *d* = 0.617, 0.441, 0.277, and 0.224 nm, respectively. As *M*_*v*_ of AAPs decreased, the (411) and (213) crystal plane diffraction peaks of COD gradually increased, whereas the ($\bar{1}01$) and (020) crystal plane diffraction peaks of COM gradually decreased. These findings indicate that the percentage of COD slowly increased, and the content of COM gradually decreased (Table 3).

Figure 6(a) also shows evident differences in the diffraction peak intensities of the crystal plane of AAP-regulated COM crystals, particularly the ratio of the diffraction intensity of the ($\bar{1}01$) crystal plane to the (020) crystal plane of COM [*I*($\bar{1}01$)/*I*(020)]. In the absence of polysaccharides (damage control group), an evident (020) plane diffraction peak of the formed COM was observed. After the addition of AAPs, not only the content of COM in the regulated crystals but also the intensity of the diffraction peak of the (020) plane of COM decreased with the decrease in *M*_*v*_ of AAPs, and *I*($\bar{1}01$)*/I*(020) increased from 0.12 in the control group to 0.73 in AAP3 (Table 3). This phenomenon was due to the easy adsorption of polyanionic AAPs with more negative charges found on the Ca^2+^-rich ($\bar{1}01$) crystal plane of COM through electrostatic interaction, which hindered the transport of Ca^2+^ from the solution to the crystal plane and inhibited crystal growth in the ($\bar{1}01$) direction [42].

The percentage of COD in each crystal was calculated using the K-value method, and the following results were obtained: control group (0%) < AAP0 (20.12%) < AAP1 (27.24%) < AAP2 (37.17%) < AAP3 (71.39%, Figure 6(b)).

#### 3.3.3. Zeta Potential

**Figure 7:**
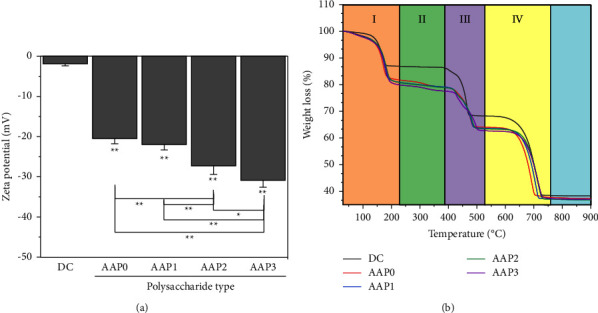
Zeta potential (a) and thermogravimetric analysis (b) of APPs-regulated CaOx. Polysaccharide concentration: 0.6 g/L. Compared with DC, ^*∗*^*p* < 0.05; ^*∗∗*^*p* < 0.01.

Figure 7(a) shows the zeta potential of CaOx crystals regulated by each AAP (0.6 g/L). The zeta potential of the crystal obtained in the absence of polysaccharides was −1.9 mV, whereas that regulated in the presence of AAPs was between −20.5 and −30.9 mV. The AAP3 group showed the largest absolute value of the zeta potential (−30.9 mV). When the charge density of the crystal surface increased, the repulsion force among crystals increased, and the aggregation degree decreased. Therefore, AAP3, which had the smallest *M*_*v*_, attained the best effect on inhibiting crystal aggregation.

#### 3.3.4. Thermogravimetric Analysis (TGA) of Crystals

The decomposition weight loss of COM (CaC_2_O_4_·H_2_O) reached 12.33% (CaC_2_O_4_·H_2_O lost water), 19.18% (CaC_2_O_4_ lost CO), and 30.13% (CaCO_3_ lost CO_2_) [43]. The decomposition of crystals without the addition of polysaccharides also proceeded through three stages (DC group in Figure 7(b)), and the weight loss rates were 13.07%, 18.62%, and 29.89% (corresponding to stages I, III, and IV, respectively; Figure 7(b)), consistent with the theoretical weight loss rate of COM. This finding indicates that crystals formed without the addition of polysaccharides were COM.

The TGA curves of AAP-regulated CaOx crystals were considerably different from those of the DC group, and such finding was attributed to the simultaneous formation of COM and COD in AAP-regulated crystals. In addition, AAPs may be adsorbed to the formed crystals. With decrease in the *M*_*v*_ of AAPs, the weight loss rate of stage I (30°C–220°C) AAP-regulated crystals gradually increased (18.26%–20.28%, Table 4). Consistently, the proportion of COD in crystals increased (Figure 6(b)) because the COD crystals had more crystalline H_2_O molecules than the COM crystal and lost more weight in stage I. The TGA results are consistent with those of XRD and SEM.

The crystals in the DC group did not show thermal weight loss in stage II (220°C–400°C), whereas the four AAP-regulated crystals presented weight loss behavior at this temperature range. As *M*_*v*_ of AAPs decreased from 31.52 kDa to 3.34 kDa, the weight loss percentage increased from 3.06% to 8.95% (Table 4). The weight loss at this stage was attributed to polysaccharide decomposition [44], which indicates that the proportion of AAPs adsorbed into the crystal increased as *M*_*v*_ of AAPs decreased; that is, the interaction of polysaccharides with the crystal increased. Gomes et al. [45] showed that polysaccharides can increase the stability of COD crystals to values higher than that of COM crystals, which is not only related to the charge effect but also to the charge distribution on the polysaccharide chain. Polysaccharides with low *M*_*v*_ had a low degree of entanglement among the main chains, a loose structure, and strong hydrogen bonds. In addition, polysaccharides with low *M*_*v*_ had more exposed −COOH and great degree of freedom. Therefore, compared with high-*M*_*v*_ polysaccharides, low-*M*_*v*_ polysaccharides had more extensive binding sites with CaOx.

### 3.4. Cytotoxicity of CaOx Crystals Regulated by Polysaccharides

#### 3.4.1. Cell Viability

**Figure 8:**
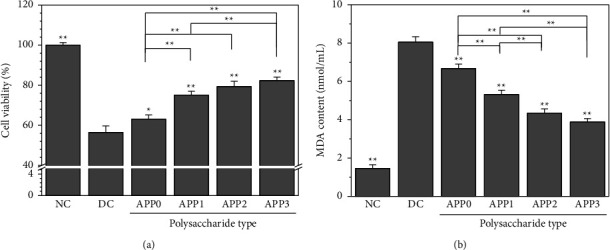
Changes of cell viability (a) and MDA content (b) of normal HK-2 cells treated with AAPs-regulated CaOx crystals. Crystal concentration: 200 *μ*g/mL; treatment time: 6 h; NC: cell group of crystal free control group; DC: crystal group formed in the absence of polysaccharide. Compared with DC, ^*∗*^*p* < 0.05; ^*∗∗*^*p* < 0.01.

As shown in Figure 8(a), CaOx crystals formed without additional polysaccharides (DC group) reduced the viability of HK-2 cells (NC group) to 56.5%. On the contrary, the cytotoxicity of the AAP-regulated crystals was reduced, and their cell viability ranged between 63.1% and 82.3%. The cell viability of the AAP3 group was 82.3% ± 1.8%, and thus the cell mortality was approximately 17.7%. The cytotoxicity of regulated crystals also decreased with the decrease in *M*_*v*_ of AAPs.

#### 3.4.2. Changes in Malondialdehyde (MDA) Content

The degree of damage to the membrane system can be indirectly assessed through the detection of MDA release on membrane lipid peroxidation products [46].  Figure 8(b) shows that the content of MDA released by the damaged crystal group was 3.89–8.05 nmol/mL, which is substantially higher than that of the control group (2.46 nmol/mL), which implies that the cell membrane was damaged by the oxidation of crystals.

#### 3.4.3. Detection of Cell Mortality by Propidium Iodide (PI) Staining

The fluorescent dye PI can specifically bind to DNA in the cell nucleus. PI cannot stain nuclei with a good membrane integrity. However, given the greatly increased membrane permeability of late apoptotic cells and dead cells, PI can penetrate the membrane of these cells and bind to DNA in the nucleus, staining it red. The more cells are stained by PI, the more cells are in the late stage of apoptosis or necrosis.

**Figure 9:**
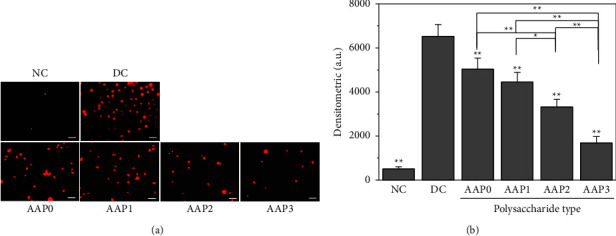
PI staining results of AAP-regulated CaOx crystals after 6 h of interaction with normal HK-2 cells. (a) Fluorescence microscope, scale: 50 *μ*m; (b) relative fluorescence intensity. Crystal concentration: 200 *μ*g/mL; time: 6 h; NC: cell group of crystal-free control group; DC: crystal group formed in the absence of polysaccharide. Compared with DC, ^*∗*^*p* < 0.05; ^*∗∗*^*p* < 0.01.

As shown in Figure 9, two nuclei in NC cells were stained red, which indicates their good condition. However, the number and degree of PI staining in DC crystals were increased remarkably. The number of cells stained red in the AAP-regulated groups was less than that in the DC group but notably more than that in the NC group (Figure 9(a)). Under the same field of view, the number of nuclei stained red by PI was in the order of AAP0 > AAP1 > AAP2 > AAP3 (Figure 9(b)), which indicates that the toxicity of crystals regulated by AAP0 to HK-2 cells was greater than that of degraded small-*M*_*v*_ polysaccharides.

#### 3.4.4. Detection of HA Molecules on the Cell Surface

**Figure 10:**
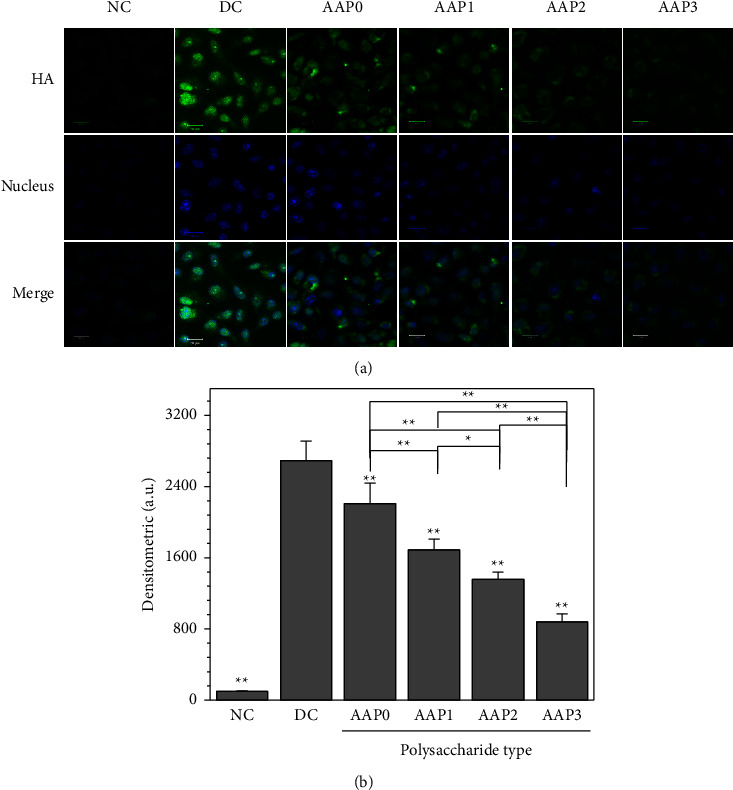
Effects of AAP-regulated CaOx crystals on HA expression in HK-2 cells. (a) Fluorescence microscopy; (b) relative fluorescence intensity. Crystal concentration: 200 *μ*g/mL; treatment time: 6 h; NC: cell group of crystal-free control group; DC: crystal group formed in the absence of polysaccharides. Compared with DC, ^*∗*^*p* < 0.05; ^*∗∗*^*p* < 0.01.

The expression of HA in renal epithelial cells is considered a cellular response after epithelial injury. The expression of negatively charged HA molecules increases the adhesion of crystals onto the cell surface and promotes the formation of kidney stones.  Figure 10 shows the difference in HA expression on the cell surface caused by CaOx and regulated by different AAPs. The intensity of green fluorescence indicates the level of HA expression. Compared with the control group, the fluorescence intensity caused by the DC group crystal formation in the absence of polysaccharides was the largest (Figure 10(a)), and the fluorescence intensity of HA caused by AAP-regulated groups had the following order: AAP0 > AAP1 > AAP2 > AAP3 (Figure 10(b)).

## 4. Discussion

### 4.1. Low *M*_*v*_ Indicates a Strong Antioxidant Activity

The antioxidant activity of polysaccharides is affected by many factors, such as monosaccharide composition, *M*_*w*_, solubility, conformation, and functional group content [47]. Given that the structure of each AAP in this paper showed no evident change, *M*_*v*_ was the main factor affecting the antioxidant activity of polysaccharides. AAP3 with a relatively low Mv had a better antioxidant activity, which is positively correlated with its hydrogen supply capacity [47]. Compared with high-*M*_*v*_ polysaccharides, low-*M*_*v*_ polysaccharides had a shorter main chain, lower degree of interchain entanglement, looser structure, and stronger hydrogen bonds. Thus, low-*M*_*v*_ polysaccharides had more free hydroxyl groups and stronger capability to terminate the free radical chain reaction. Given that the active groups (−COOH, etc.) of low *M*_*v*_ polysaccharides were more exposed and had greater degree of freedom, the steric hindrance was small, and the chelating capability was enhanced during the chelation with Fe^2+^. Specifically, as shown in Figure 4, the following detection indexes revealed the concentration-dependent trend of AAP3: At the concentration of 3.0 mg/mL, compared with AAP0, the ·OH free radical-scavenging capability of AAP3 was about 1.4 times higher, the reducing capacity was approximately 1.9 times higher, and the chelating Fe^2+^ capacity was around 5.0 times higher. The following detection indicators plateaued when the concentration reached 1.0 mg/mL: Compared with AAP0, the DPPH and ABTS free radical-scavenging capability of AAP3 were around 2.2 and 2.3 times higher, respectively. Zhang et al. [48] extracted and isolated four polysaccharide components (with *M*_*w*_ = 10.9 × 10^3^, 3.69 × 10^3^, 1.83 × 10^3^, and 1.72 × 10^3^ kDa, respectively) from *Pinus koraiensis*, and they showed dose-dependent scavenging effects on hydroxyl radicals and ABTS radicals, with IC50 values of 122.2, 2.6, 3.0, and 2.9 mg/mL, respectively. Zhang et al. [49] used microwave degradation to degrade *Polygonatum sibiricum* polysaccharides (PSP0) with a *M*_*w*_ of 2.99 × 10^5^ Da to 2.33 × 10^3^ Da (PSP1) and observed that the antioxidant activity of degraded PSP1 was eight times higher than that of PSP0 after the detection of their capability to scour DPPH free radicals.

### 4.2. AAP-Regulated Growth of CaOx Crystal

#### 4.2.1. Induced COD Formation

The results of XRD analysis (Figure 6(a)) show that the degraded AAPs can effectively inhibit the growth of COM and induce the formation of COD crystals. In addition, the induced COD content increased with the decrease in *M*_*v*_ of AAPs (Figure 6(b)), among which AAP3 remarkably regulated the formation of COD crystals. The percentages of COD in the crystals of each group were observed in the following order: DC group (0%) < AAP0 (20.12%) < AAP1 (27.24%) < AAP2 (37.17%) < AAP3 (71.39%; Figure 6(b)).

**Figure 11:**
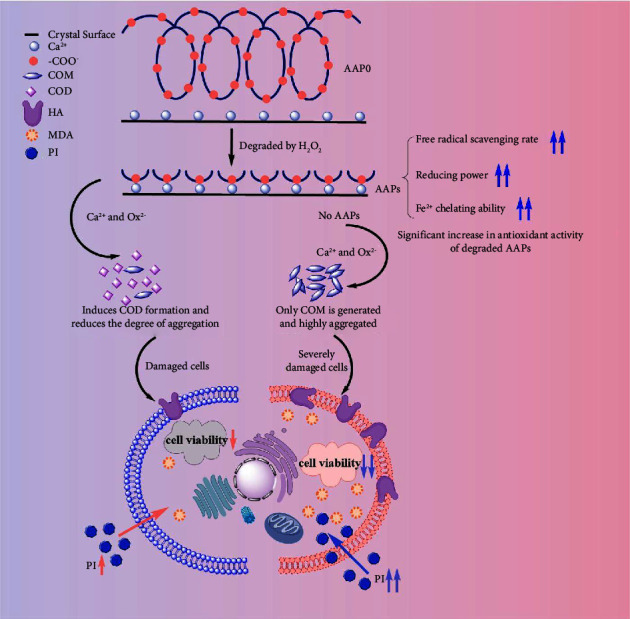
Mechanism diagram of AAPs regulate CaOx crystal growth and the regulated crystals to damage HK-2 cells.

Figure 11 shows the regulatory mechanism of AAPs on CaOx crystals. AAPs contained a large number of −COOH groups, which can complex with Ca^2+^. After the AAPs were complexed with Ca^2+^, the concentration of Ca^2+^ on the surface of AAP molecules increased rapidly. Based on the logistic regression analysis performed by Daudon et al. [50], the increase in Ca^2+^ concentration promoted the formation of COD.

#### 4.2.2. Inhibition of Crystal Aggregation

Given the large amount of negative charges, AAPs can be adsorbed onto the surface of CaOx crystals through electrostatic interaction, which led to the increase in negative charges of crystals and the enhancement of their mutual repulsion. In addition, the aggregation of crystals was inhibited. Compared with high-*M*_*v*_ polysaccharides, AAP3 induced more negative zeta potential on the crystal surface (Figure 7(a)); that is, the repulsion among crystals was the largest. Thus, AAP3 had the strongest capability to inhibit crystal aggregation (Figure 5).

### 4.3. Differences in the Cytotoxicity of AAP-Regulated CaOx Crystals

CaOx crystals induced oxidative stress in HK-2 cells, which caused cell damaged and increased crystal adhesion. The cytotoxicity of CaOx crystals is closely related to their concentration, size, crystal phase (COM or COD), morphology, and doping [51].

#### 4.3.1. Greater Cell Adhesion to COM than to COD

Given the different proportions of COM and COD in the AAP-regulated crystals, the cytotoxicity of the regulated crystals varied. Although COM and COD differed by only one bound water, they showed considerably different characteristics [52]. Given the high surface positive-charge density, COM had a higher affinity to injured cells than COD. Lieske et al. [53] observed that COM crystals bound to apical microvilli on the surface of monkey renal epithelial cells (BSC-1) in a few seconds, and the apical microvilli migrated on the surface of crystals; that is, COM crystals can rapidly adhere to the apical surface of BSC-1 cells and promote their retention in the kidney. Sheng et al. [5] used biologically relevant functional groups as probes and reported the higher adhesion strength of COM than COD. Wang et al. [52] revealed that when HK-2 cells adhered to COM and COD crystals, their adhesion kinetics were substantially different. At the initial stage of attachment, some COD crystals can be temporarily outside of the cell because the cell adhesion to COM was stronger than that to COD; that is, only the adhesion of COM crystals occurred. The authors suggest that this phenomenon may prevent urolithiasis.

Therefore, the toxic effect of CaOx crystals on cells can be reduced by promoting the conversion of COM to COD. The results of this paper show that the percentage of COD in AAP-regulated CaOx crystals is directly related to *M*_*v*_ of polysaccharides. AAP0 and AAP3 regulated 20.12% and 71.39% of COD, respectively. Therefore, the crystals regulated by AAP3 had the least toxicity to cells (Figures 8(a) and 9), lowest MDA content (Figure 8(b)), and lowest HA expression (Figure 10).

#### 4.3.2. Effect of Doped Polysaccharides on Crystals

The cytotoxicity of AAP-regulated crystals was attenuated because of the adsorption or incorporation of polysaccharides into the crystals (Figure 7(b)). Our previous studies have shown that the proportions of sulfated *Porphyra yezoensis* polysaccharides with different sulfate contents incorporated into the crystals were between 6.42% and 8.38% [54], whereas those of carboxymethylated *Poria cocos* polysaccharides with different carboxyl contents incorporated into the crystals were in the range of 9.78%–20.52% [55]. In addition, the smaller the *M*_*v*_, the greater the proportion of polysaccharides doped into the crystals. In this paper, AAP3, which had the smallest *M*_*v*_, exhibited the strongest biological activity and largest binding degree with CaOx crystals. The proportion of adsorption to crystals (8.95%) was higher than that of the other three AAPs (3.06%–6.02%, Table 4). Thus, the CaOx crystals regulated by AAP3 presented the lowest cytotoxicity.

#### 4.3.3. Effect of Aggregation State, Size, and Morphology of Crystals

The degree of sharpness of the induced crystals decreased from the DC group to the AAP0, AAP1, AAP2, and AAP3 groups (Figure 5). Given that the crystals with sharp edges and corners caused more serious damage to cells than blunt crystals [56], their cytotoxicity also increased in turn.

In addition, the sizes of AAP-regulated CaOx crystals were larger than that formed in the absence of polysaccharides, which also reduced the cytotoxicity of CaOx. Our previous study [57] showed that the toxicities of micron COD and COM crystals to Vero cells were in the following order: 1 *μ*m > 3 *μ*m > 10 *μ*m.

### 4.4. Effect of *M*_*w*_ of Polysaccharides on Their Biological Activity

The antioxidant activity and capability of APPs to promote COD crystal formation were observed in the following order: AAP0 < AAP1 < AAP2 < AAP3; that is, the smaller the *M*_*v*_, the higher the biological activity. Given the small difference in carboxyl content and molecular structure of the four AAPs, the differences in the biological activities of AAPs were primarily due to changes in their *M*_*v*_.

The natural polysaccharide AAP0 had a tighter structure and stronger intramolecular hydrogen bond because of its large *M*_*v*_, which resulted in the decreased activity of its active groups (such as −COOH). In addition, polysaccharides with large *M*_*v*_ have long overlapping chains and high steric hindrance, which result in their low bioactivity.

The biological activity of polysaccharides depends on the helical structure of the backbone and on the hydrophilic groups (hemiacetal, sulfate, carboxyl, and hydroxyl groups) on the outer surface of the helical structure [58]. Compared with high-*M*_*v*_ polysaccharides, low-*M*_*v*_ polysaccharides expose more hydrophile groups and reducing ends (hemiacetals), which are conducive to adsorption on the crystal surface; such condition results in reduced zeta potential (Figure 7(a)), increased intercrystalline repulsion, reduced toxic effects between crystals and cells (Figures 8(a) and 9), and strong biological activity [58]. Figure 11 shows the mechanistic damage effect of AAP-regulated crystals on HK-2 cells.

The results show AAP-regulated CaOx crystals by displayed weak cytotoxicity to cells and low MDA and HA expression levels with the decrease in *M*_*v*_ of AAPs (Figures 8(b) and 10). This result is consistent with that in the literature studies [59, 60]; that is, the lower the *M*_*v*_, the better the biological activity of polysaccharides. Deng et al. [59] observed that the immunoregulatory activity of *Lycium barbarum* polysaccharide (LBP) fractions on RAW264.7 macrophages was closely related to their *M*_*v*_, and that of LBP2 fraction with a *M*_*v*_ of 350 kDa was relatively limited. Sun et al. [60] evaluated the in vivo S180 tumor-bearing mouse model and in vitro peritoneal macrophage activation and reported that six degraded laver polysaccharides (*M*_*v*_ = 6.53−1002 kDa) showed significant immunomodulatory effects in varying degrees. Furthermore, the fragment with the smallest *M*_*v*_ had the strongest immunopotentiating activity.

## 5. Conclusions

Four AAPs with similar −COOH contents (4.48%) but different *M*_*v*_ of 31.52, 11.82, 5.86, and 3.34 kDa showed antioxidant capacities in vitro. AAPs can inhibit COM growth, induce COD generation, and reduce the degree of crystal aggregation. AAP-regulated CaOx crystals exhibited cytotoxicity to HK-2 cells, but they were less than the crystals formed in the absence of polysaccharides. As *M*_*v*_ of AAPs decreased, their biological activity increased. The cytotoxicity of regulated CaOx crystals is closely related to the percentage of COD, degree of crystal aggregation, and sharpness of crystal edges. AAPs, especially AAP3 in small Mv, have a potential stone prevention effect.

## Figures and Tables

**Table 1:** Degradation conditions and physicochemical properties of AAPs with different molecular weights.

Polysaccharide abbreviation	Concentration of H_2_O_2_ (%)	Intrinsic viscosity [*η*] (mL/g)	Mean molecular weights *M*_*v*_ (kDa)	–COOH content (%)
AAP0	0	38.5	31.52	4.28
AAP1	0.3	14.4	11.82	4.54
AAP2	2	7.15	5.86	4.63
AAP3	8	4.07	3.34	4.67

**Table 2:** Monosaccharide composition of degraded polysaccharide AAP3.

Polysaccharide abbreviation	*M*_*v*_ (kDa)	Carboxyl content (%)	Monosaccharide percentage (%)^*∗*^^1^							
			Glu	Man	Ara	Gal	Rha	Xyl	Fuc	Myo
AAP3	3.34	4.67	61.45	17.44	14.65	3.26	3.20	0	0	0

[^*∗*^^1^]: Glu: glucose; Man: mannose; Ara: arabinose; Gal: galctose; Rha: rhamose; Xyl: xylose; Fuc: fucose; Myo: myo-inositol.

**Table 3:** Changes of COD percentage and COM crystal plane intensity in the regulated CaOx crystals by AAPs with different molecular weights. Polysaccharide concentration: 0.6 g/L.

Polysaccharide abbreviation	COD (%)	Diffraction intensity ratio of COM crystal plane $I_{\left(\bar{1}01\right)}$/*I*_(020) _
DC	0	0.12
AAP0	20.12	0.22
AAP1	27.24	0.65
AAP2	37.17	0.70
AAP3	71.39	0.73

**Table 4:** The weight loss analysis of thermogravimetric curves of the AAPs-regulated CaOx crystals. Polysaccharide concentration: 0.6 g/L.

Polysaccharide abbreviation	Stage I (%)	Stage II (%)^*∗*^^1^	Stage III (%)	Stage IV (%)	Residue (%)
DC	13.07	0	18.62	29.89	38.42
AAP0	18.26	3.06	14.62	27.06	37.00
AAP1	19.00	5.57	11.67	26.54	37.23
AAP2	19.15	6.02	11.31	26.46	37.07
AAP3	20.28	8.95	8.03	25.52	37.22

Pure COM	13.33	0	19.18	30.13	38.36

Pure COD	21.95	0	17.07	26.83	34.15

^*∗*^^1^The weight loss of stage II refers to the decomposition of polysaccharide absorbed in the crystal, that is, the percentage of polysaccharide absorbed in the crystal.

## Data Availability

All data used to support the findings of this study are included within the article.
